# The relationships between bariatric surgery and sexual function: current evidence based medicine

**DOI:** 10.1186/s12894-020-00707-1

**Published:** 2020-10-02

**Authors:** Shengzhuo Liu, Dehong Cao, Zhengju Ren, Jinze Li, Lei Peng, Qin Zhang, Bo Cheng, Zheyu Cheng, Jianzhong Ai, Xiaonan Zheng, Liangren Liu, Qiang Wei

**Affiliations:** 1 Department of Urology, Institute of Urology, West China Hospital, Sichuan University, Chengdu, Sichuan China; 2 DepartmentofUrology, Nanchong CentralHospital, The Second ClinicalMedical College, NorthSichuanMedicalCollege (University), Nanchong, Sichuan China; 3 Department of Radiology, Chongqing Traditional Chinese Medicine Hospital, Chongqing, China

**Keywords:** Bariatric surgery, Obesity, Erectile function, Sexual function, Sex hormone, Meta-analysis

## Abstract

**Background:**

Controversy remains despite several studies have discussed the role of bariatric surgery in improving male’s sexual function. This study aims to evaluate the efficacy of bariatric surgery in promoting male’s erectile function.

**Methods:**

PubMed, EMbase, The Cochrane Library, CNKI and Clinical Trails.gov were searched from database inception to May 2019. The language of publication was limited in English. The International Index of Erectile Function (IIEF) score and Brief Male Sexual Function Inventory (BSFI) score were set as the primary outcome.

**Results:**

Eleven studies with a total of 370 patients were enrolled in this meta-analysis. The results showed significant improvement in the IIEF score (erectile function: MD = 5.33, 95% CI 4.12–6.54; intercourse satisfaction: MD = 2.57, 95% CI 1.19–3.94; orgasmic function: MD = 0.50, 95%CI 0.60–0.94; overall satisfaction: MD = 1.67, 95% CI 0.78–2.56; sexual desire: MD = 1.27, 95% CI 0.61–1.93; total erectile function: MD = 7.21, 95% CI 4.33–10.10) and the BSFI score (erection: MD =2.53, 95% CI 2.39–2.67; ejaculation: MD = 1.40, 95% CI 1.28–1.51; desire: MD =1.40, 95% CI 1.32–1.49; problem assessment: MD = 2.20, 95% CI 2.06–2.34; sexual satisfaction: MD = 0.70, 95% CI 0.60–0.76) in obese individuals after bariatric surgery.

**Conclusions:**

This systematic review and meta-analysis indicated that bariatric surgery could be effective in promoting males’s sexual function for obese individuals.

## Background

Erectile dysfunction, one of the most complained problems during sexual intercourse, is defined as the inability to achieve or maintain an erection sufficient for satisfactory sexual performance [1], which has been paid more and more attention for its` association with poor quality of male’s sexual life [2] and high prevalence. Epidemiological studies indicated that in 1995 erectile dysfunction (ED) affected over 150 million men worldwide and the prevalence will reach 322 million by 2025 [3], which could be a great healthy burden to the society for its high prevalence in the general population and tight association with life quality impairment [4].

Obesity, especially morbid obesity, is associated with a broad range of medical and psychosocial comorbidities which impose a negative effect on patients’ lives and health-care systems, including non-insulin dependent diabetes mellitus, coronary heart disease, stroke and hypertension [5–8]. Evidence from a large number of studies have shown relationships between obesity and derangements in sexual function [9–11]. It was reported that the occurrence of ED was higher in obese males than in those of normal weight [12]. Sarwer et al. [11] demonstrated in their study that 36% of men seeking bariatric surgery reported erectile dysfunction. Moore et al. [13] reported in their study 45% of obese men who seek weight reduction met diagnostic criteria of erectile dysfunction. Laumann et al. [4] reported in the United States that the prevalence of sexual dysfunction in general male population was 31%.

Several managements have been introduced to deal with obesity. Dietary intervention, medications, physical exercise are widely used in case of mild and moderate obesity [14]. Meanwhile, bariatric surgery has become the most effective treatment strategy for morbid obese patients which can bring great weight reduction and is expected to ameliorate the comorbidities [15, 16]. The bariatric surgical procedures, including gastric bypass, vertical sleeve gastrectomy, and biliopancreatic diversion, are recommended for class 2 obesity associated with comorbidities and for class 3 obesity (body index [BMI] 35–39.9 kg/m^2^ and > 40 kg/m^2^;respectively) [17].

As an effective procedure in promoting weight loss and improving obesity-related physical comorbidities, bariatric surgery is now widely performed on obese patients [18–21]. A number of studies have studied the effect of bariatric surgery on male’s sexual function while the results are inconsistent. Significant improvement was observed in some studies but not in the other studies. Hence, we performed the systemic review and meta-analysis to investigate the efficacy of bariatric surgery in promoting male’s sexual function.

## Methods

Before conducting a systematic literature search, we formulated a protocol to standardize the process of literature review and data extraction which is agreed by all the authors.

### Inclusion and exclusion criteria

Two independent reviewers separately assessed each study based on the inclusion and exclusion criteria to determine whether the study is eligible. Studies meeting the following criteria were included: (1) self-control study, (2) randomized controlled trials which use bariatric surgery as intervention, (3) at least one of the erectile function assessment score system (IIEF score and BSFI score**)** was assessed and reported. For exclusion, we eliminated studies which met the following criteria: (1) only females were recruited as the study subjects, (2) either perioperative data or postoperative data was missing or not recorded, (3) reviews, letters, technical reports, case reports, comments and case series studies. Publications based on the same study (e.g. same authors, institutions, period of study) were discussed, and only the best-quality study was included.

### Search strategy

We searched PubMed, EMbase, The Cochrane Library, CNKI and Clinical Trails.gov for English-language studies published before May 2019. Search terms included bariatric, laparoscopic Roux-and-Y gastric bypass, gastric bypass surgery, obesity, erectile dysfunction and sexual function. Reference lists of related articles, reviews, meta-analysis and other types of articles were also searched manually to identify additional eligible studies.

### Study selection

Two reviewers independently reviewed the titles and abstracts of all retrieved studies. The full texts of studies which are deemed to be relevant studies were reviewed in details after the initial screening.

### Data extraction

For each included study, we extracted data by two independent reviewers. Parameters including leading author, country, patient composition, methods for recruitment, baseline IIEF (or BSFI) score, postoperative IIEF (or BSFI) score and postoperative time point for SF assessment were recorded. We used the five-question International Index of Erectile Function (IIEF) score and BFSI score before and after bariatric surgery as the main reference index to evaluate the erectile function.

### Quality assessment

We used a modified version of the methodological index for non-randomized studies’ (MINORS[ [22, 23]: Table 1) checklist to assess methodological quality of the included systematic reviews. The checklist contains the following 8 aspects: (1) a clearly stated aim, (2) inclusion of consecutive patients, (3) prospective collection of data, (4) endpoints appropriate to the aim of the study, (5) unbiased assessment of the study endpoint, (6) follow-up period appropriate to the aim of the study, (7) loss to follow up less than 5%, (8) prospective calculation of the study size.

**Table 1:** Quality assessment for all of the included studies

Included studies	1. A clearly stated aim	2. Inclusion of consecutive patients	3. Prospective collection of data	4. Endpoints appropriate to the aim of the study	5. Unbiased assessment of the study endpoint	6. Follow-up period to the aim of the study	7. Loss to follow up less than 5%	8. Prospective calculation of the study size	Total
Sarwer et al. [10]	2	2	1	2	1	2	2	1	13
Reis et al. [24]	2	2	2	2	1	2	2	1	14
Ranasinghe et al. [25]	2	2	2	2	2	1	2	1	14
Mora et al. [26]	2	1	2	2	2	2	2	1	14
Li et al. [27]	2	2	1	2	1	2	2	1	13
Groutz et al. [28]	2	1	2	2	2	1	2	1	13
Efthymious et al. [14]	2	2	2	2	2	2	2	1	15
Aleid et al. [29]	2	2	2	2	2	2	2	1	15
ARAÚJO et al. [30]	2	1	1	2	2	2	2	1	13
Dallal et al. [31]	2	2	2	2	1	2	2	2	15
Goitein et al. [32]	2	1	2	2	1	2	2	1	13

### Data analysis

We performed the meta-analysis by using the Cochrane Review software (Review manager v.5.3 for windows). To analyze the categorical variables, the Cochran-Mantel-Haenszel test was conducted and the forest plots was performed to show the results. Heterogeneity analysis uses the I^2^ test to define the statistic heterogeneity between each study. The heterogeneity was deemed to be acceptable and fixed model was used if the I^2^ was less than 50%, while the heterogeneity was considered high and the random model was used if the I^2^ was greater than 50%.

## Results

### Search results and characteristics of the included studies

**Fig. 1:**
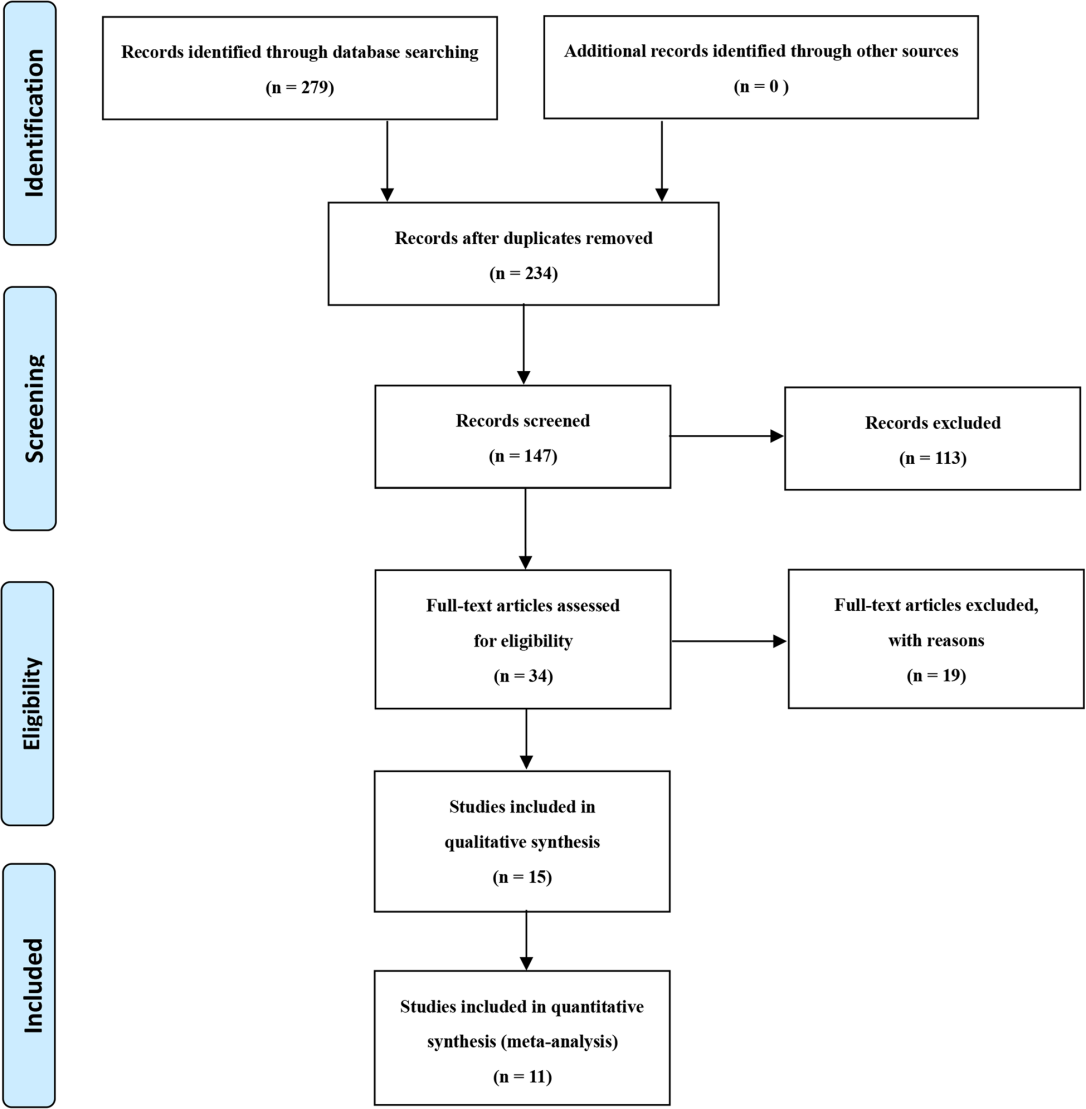
Preferred Reporting Items for Systematic Reviews and Meta-Analyses (PRISMA) 2009 flow diagram [33]

We identified 279 studies totally. After the initial screening through the titles and abstracts, we assessed the full-text of articles eligible for detailed assessment. Finally, 11 studies met the inclusion criteria and were included in this study. The flowchart of the procedure is shown in Fig. 1. The details of the included studies are presented in Table 2.

**Table 2:** Characteristics of eligible studies

Study (Included studies)	Country	Year	Number of Patients	Age, years (± SD)	Pre-op BMI, kg/m^2^ (± SD)	Follow-up (months)	Surgery types	Study types	Sexual function index
Sarwer et al. [10]	UK	2012	31	48	45.10	12	RYGB	PS	IIEF
Reis et al. [24]	Brazil	2009	10	36.7 ± 11.5	31.0 ± 5.3	24	RYGB	PS, RCT	IIEF
Ranasinghe et al. [25]	UK	2009	20	52.8 ± 9.33	7.3 ± 12.67	12	LGB	RS	IIEF
Mora et al. [26]	Spain	2013	39	43.5 ± 10.25	30.18 ± 5.04	12	BS	PS	IIEF
Li et al. [27]	China	2014	39	45.2 ± 10.0	41.2 ± 8.5	12	RYGB	RS	IIEF
Groutz et al. [28]	Israel	2016	39	40.7 ± 12.4	42.8 ± 5.6	3	LSG	PS	IIEF
Efthymious et al. [14]	Greece	2014	30	37.3 ± 9.6	50.66 ± 7.9	12	SG/RYGB/BPD	PS	IIEF
Aleid et al. [29]	UK	2016	30	48.9 ± 7.0/44.1 ± 6.9^#^	46.8(37.9〜61.9)/47.8 (37.1〜69.5)^#^	6	LGB/LSG	PS	IIEF
ARAÚJO et al. [30]	Brazil	2009	21	20〜50	36〜89	6	BS	PS	IIEF
Dallal et al. [31]	America	2008	97	47.9 (19〜75)	51.4 (36〜89)	19	RYGB	PS	BSFI
Goitein et al. [32]	Israel	2015	14	44.8 ± 13.9	40.9 ± 4.2	6〜7	LSG、LRYGB	PS	BSFI

### Results of each study

Results of each included study were illustrated in Table 3.

**Table 3:** Results of each study

Included studies	Outcomes	Preoperative	Postoperative	***P*** value	Included studies	Outcomes	Preoperative	Postoperative	***P*** value
		Mean SD	Mean SD				Mean SD	Mean SD	
**Sarwer et al.** [10]	IIEF: erectile function	19.9 (11.6)	21.3 (10.4)	0.910	**Aleid et al.** [29]	IIEF: erectile function^#1^	13.0	–	<0.02
	IIEF: orgasm function	7.1 (3.9)	7.2 (4.0)	>0.99		IIEF: orgasm function	8.0	–	<0.004
	IIEF: sexual desire	6.4 (2.5)	7.6 (2.2)	0.058		IIEF: sexual desire	6.0	–	<0.002
	IIEF: intercourse satisfaction	7.3 (5.6)	8.5 (5.2)	0.673		IIEF: intercourse satisfaction	6.0	–	<0.004
	IIEF: overall satisfaction	6.1 (2.8)	7.0 (2.6)	0.266		IIEF (total)	64.0	66.5	<0.001
**Reis et al.** [24]	IIEF-5	19.7 ± 6.6	23.0 ± 2.3	0.0469	**ARAÚJO et al.** [30]	[24]	24.00 ± 5.99	27.85 ± 2.45	0.005
**Ranasinghe et al.** [25]	IIEF (total)	51.36 (22.17)	48.17 (25.34)	0.712		IIEF: orgasm function	9.14 ± 1.27	9.38 ± 0.97	0.234
**Mora et al.** [26]	IIEF: erectile function	21.95 ± 8.03	25.74 ± 6.63	0.002		IIEF: sexual desire	7.66 ± 2.03	8.61 ± 1.32	0.061
	IIEF: orgasm function	8.08 ± 2.30	8.15 ± 2.54	0.843		IIEF: sexual relations	10.09 ± 2.58	11.80 ± 2.20	0.005
	IIEF: sexual desire	7.54 ± 1.79	8.15 ± 1.33	0.013		IIEF: overall satisfaction	7.09 ± 1.48	8.09 ± 1.13	0.031
	IIEF: intercourse satisfaction	9.67 ± 4.19	10.67 ± 3.62	0.083	**Dallal et al.** [31]	BSFI: sex drive	3.9 ± 0.3	5.3 ± 0.3	<0.001
	IIEF: overall satisfaction	7.62 ± 2.40	8.49 ± 2.15	0.047		BSFI: erection	6.4 ± 0.5	8.9 ± 0.5	<0.001
	IIEF (total)	54.85 ± 16.59	61.21 ± 14.10	0.006		BSFI: ejaculation	4.9 ± 0.4	6.3 ± 0.4	<0.001
**Li et al.** [27]	IIEF-5	17.3 ± 5.5	23.8 ± 6.1	0.004		BSFI: problem assessment	7.4 ± 0.5	9.6 ± 0.5	<0.001
**Groutz et al.** [28]	IIEF: erectile function	22.7 ± 7.2	26.1 ± 6.5	0.02		BSFI: sexual satisfaction	1.6 ± 0.2	2.3 ± 0.2	O.002
	IIEF: orgasm function	8.5 ± 2.8	9.2 ± 1.9	NS	**Goitein et al.** [32]	BSFI: desire	6.1 ± 1.6	7.8 ± 2.7	0.018
	IIEF: sexual desire	7.8 ± 2.1	8.4 ± 1.5	NS		BSFI: erection	4.5 ± 0.8	12 ± 3.6	0.043
	IIEF: intercourse satisfaction	9.5 ± 4.2	11.5 ± 3	0.01		BSFI: ejaculation	9 ± 1.3	8.3 ± 2.6	0.315
	IIEF: overall satisfaction	7.9 ± 2.5	8.9 ± 1.3	0.02		BSFI: problem assessment	11.3 ± 3.7	11.8 ± 4	0.042
**Efthymious et al.** [14]	IIEF: erectile function	18.63 ± 9.63	24.85 ± 8.17	0.002		BSFI: sexual satisfaction	2.8 ± 0.8	4.1 ± 1.1	0.0006
	IIEF: orgasm function	8.33 ± 2.64	8.59 ± 2.90	0.727		BSFI (total)	40.2 ± 9.2	43.9 ± 12	0.064
	IIEF: sexual desire	6.29 ± 2.27	8.18 ± 1.92	0.001					
	IIEF: contact satisfaction	5.15 ± 5.26	11.07 ± 4.51	<0.001					
	IIEF: total satisfaction	5.29 ± 2.91	8.59 ± 1.32	<0.001					

#1(ED group)

Sarwer et al. [11] conducted a prospective cohort study with 32 men who underwent a Roux-en-Y gastric bypass enrolled, and investigated the sexual function of individuals by using the International Index of Erectile Functioning (IIEF) and sex hormones. The results showed that there was no significant change of the sexual function from the baseline except of overall satisfaction at prospective year 3 (*P* = 0.008), though the men reported improvements in sexual functioning.

On the contrary, the prospective cohort study performed by Reis et al. [24]. studied 10 morbidly obese men to measure the degree of sexual function change after life style modifications (exercise and diet) for 4 months and subsequently gastric bypass. Bariatric surgery induced weight loss was found to improve erectile function quality in the research.

Ranasinghe et al. [25]. investigated the effect of weight loss and laparoscopic gastric banding surgery on sexual function among 20 obese men. The results suggested that the IIEF score achieved significant improvement after surgery while they also observed worsen erectile index (*P* = 0.005) and orgasmic function (*P* = 0.002).

In a prospective study, Mora et al. [26]. found the IIEF score increased significantly at 1 year after bariatric surgery in 39 patients. Li et al. [27]. conducted a retrospective cohort study and observed significant improvement in IIEF score after RYGB. Groutz et al. [28]. enrolled 39 consecutive obese men who underwent laparoscopic sleeve gastrectomy to investigate the effect of bariatric surgery on male’s sexual function in a prospective study. The IIEF were recorded preoperatively and postoperatively. The results demonstrated that male’s sexual function, including erectile function, overall intercourse satisfaction and overall satisfaction, was significantly improved after surgery. In another prospective study performed by Efthymious et al. [14], they found the bariatric surgery could significantly improve sexual function, especially in the first 6 months after surgery. In the prospective study by Aleid et al. [29]., significant improvement in male’s erectile function after surgery was also reported.

Araujo et al. [30]. evaluated the effects of Fobi-Capella gastroplasty on quality of male’s sexual life and observed favorable changes in sexual function postoperatively.

Dallal et al. [31]. compared the Brief Male Sexual Function Inventory (BSFI) before and after gastric bypass surgery to measure its effect on the sexual function in morbidly obese man. In summary, the patients reported a significant improvement in all domains of BSFI scores postoperatively.

Goitein et al. [32]. found the BSFI scores increased but did not reach statistical significance (*P* = 0.08). However, general satisfaction, erection and desire were significantly improved after surgery.

### Meta-analysis

**Fig. 2:**
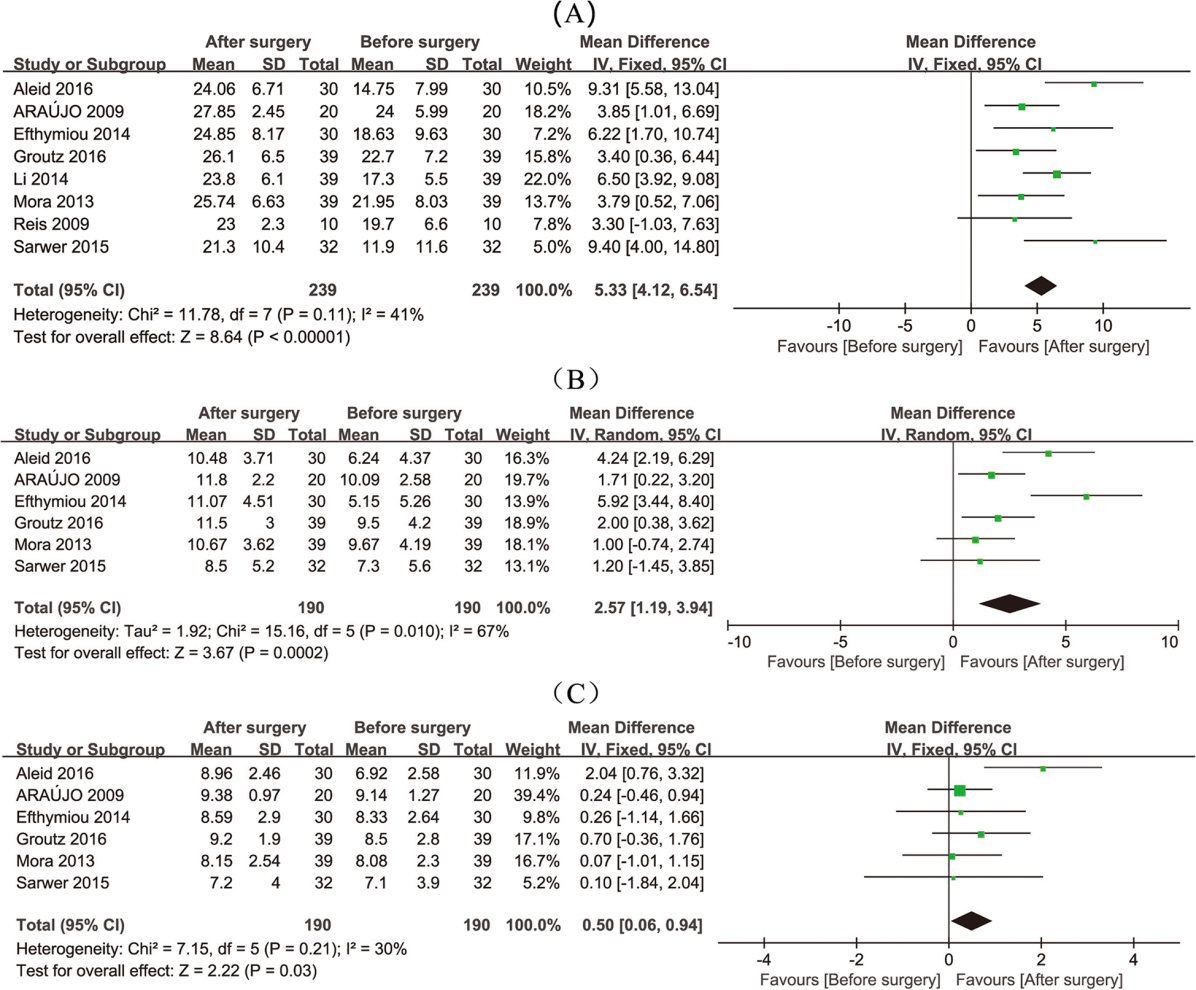
Meta-analysis on the efficiency of bariatric surgery in promoting male’s sexual function. Comparison of Erectile Function domain of IIEF (**a**), Intercourse Satisfaction domain of IIEF (**b**), and Orgasmic Function domain of IIEF (**c**)

**Fig. 3:**
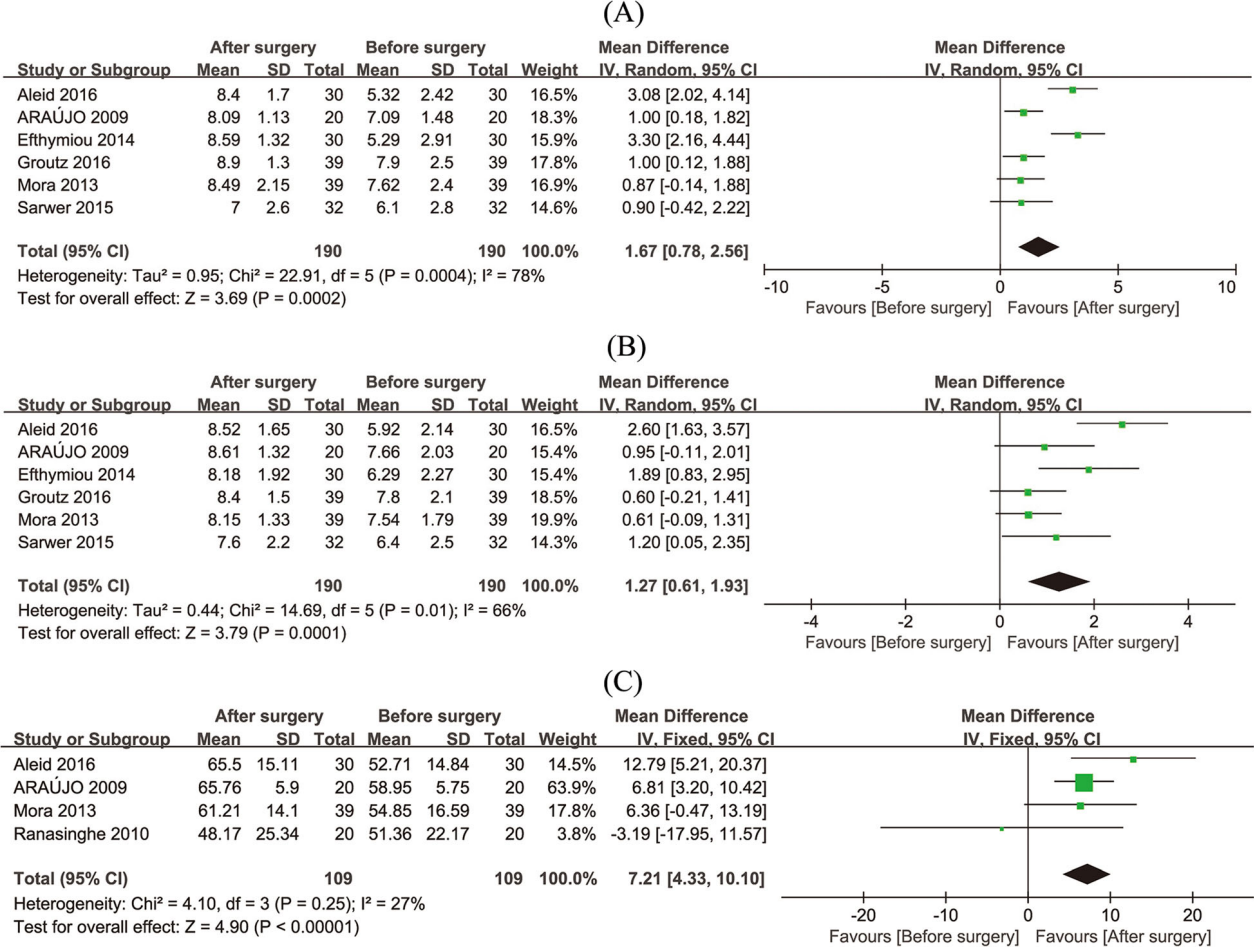
Meta-analysis on the efficiency of bariatric surgery in promoting male’s sexual function. Comparison of Overall Satisfaction domain of IIEF (**a**), Sexual Desire domain of IIEF (**b**), and Total Erectile Function domain (**c**)

The analysis was based on 370 patients from 11 studies. From the results of studies using IIEF as the index measuring erectile function [11, 14, 24, 26–30], the postoperative erectile function was significantly improved (Fig. 2a, 95% CI 4.12–6.54, *p* < 0.001). Improvement was also observed in the intercourse satisfaction (Fig. 2b, 95% CI 1.19–3.94, *p* = 0.0002), orgasmic function (Fig. 2c, 95%CI 0.60–0.94, *p* = 0.03), overall satisfaction (Fig. 3a, 95% CI 0.78–2.56, p = 0.0002), and sexual desire (Fig. 3b, 95% CI 0.61–1.93, *p* = 0.0001). Meanwhile, total erectile function showed a 7.21-point increasement in these studies (Fig. 3c, 95% CI 4.33–10.10, *p* < 0.001). In studies [34, 35] which used BSFI as the index measuring erectile function, favorable results showed improvement in erection (95% CI 2.39–2.67, *p* < 0.001), ejaculation (95% CI 1.28–1.51, *p* < 0.001), desire (95% CI 1.32–1.49, *p* < 0.001), problem assessment (95% CI 2.06–2.34, *p* < 0.001) and sexual satisfaction (95% CI 0.60–0.76, *p* < 0.001).

## Disscussion

Obesity has been a worldwide health problem and can adversely affect sexual functioning. In the Massachuster Male Aging Study [31], the average prevalence of erectile dysfunction in men who were not overweight was 13% while the altered prevalence in those who were overweight at baseline was 22%. Nowadays, the bariatric surgery has become the predominant treatment for morbid obesity and is reported to be the most effective option for weight loss in the severely obese people who have excessive fat accumulation [36]. The previous studies have documented favorable clinical outcomes after bariatric surgery which can lose weight effectively and bring significant reduction in the disease-specific risk of death [18]. However, relevant researches, investigating the correlation between the bariatric surgery and male’s sexual function, are insufficient. Meanwhile, the definitive causal link between bariatric surgery and male’s sexual function has not been widely studied. Moreover, some relevant studies which investigated the effect of bariatric surgery on male’s sexual function of morbidly obese patients were somewhat limited by lacking of sample sizes, indefinite results, and are of low credibility. Thus, we performed the meta-analysis to make a comprehensive analysis of those studies in order to investigate whether bariatric surgery contributes to improvements on male’s erectile function.

To our knowledge, this is the first meta-analysis to evaluate the effect of bariatric surgery on male’s sexual function with both IIEF scores and BSFI scores enrolled for comparison. Though a previous meta-analysis, by Glina et al. [34], was also performed to assess the effect of bariatric surgery on male’s erectile function change, they only discussed the IIEF change and only 7 studies were enrolled for analysis.

The meta-analysis results indicate that bariatric surgery presents conspicuously effective improvements on male’s sexual function by comparing IIEF scores, including erectile function, intercourse satisfaction, overall satisfaction, orgasmic function, sexual desire, total erectile function and BSFI scores, including erection, ejaculation, problem assessment, sexual satisfaction. According to Aleid et al. [29], the male’s sexual function improvements after bariatric surgery could be reflected by increasement of all the IIEF domains. This could be in accordance with the study performed by Efthymious et al. [14] and other researchers [24, 26, 27]. In contrast, in studies undertaken by Ranasinghe et al. [25] and Sarwer et al. [11], which also used IIEF to assess male’s sexual function, they found no significant improvement of erectile function among obese men undergoing bariatric surgery. Dallal et al. [31], retrospectively studied 97 obese men who had undergone gastric bypass surgery with a mean postoperative follow-up length of 19 months. In this study, they found that scores of all domains of the BSFI including sexual drive, erectile function, ejaculatory function and sexual satisfaction improved postoperatively. Meanwhile, the postoperative BSFI scores approached the reference controls. This is in accordance with our findings which indicated significant improvements on all domains of BFSI score.

However, the underlying mechanism of obesity-related sexual dysfunction has not been clearly elucidated. Previous studies suggested that psychological and social appearance, such as body image, depression and so on, have a negative impact on self-esteem and the tendency of avoidance and initiation to sexual behavior [35]. Negative effect of the comorbidities in obese people (diabetes, hypertension, metabolic syndrome, etc.) have already been clearly associated with sexual dysfunction [10, 31, 37]. A correlation of sex hormones to sexual function has also been confirmed, which was believed to be associated with obesity induced sexual dysfunction [38, 39]. Meanwhile, a number of biological mechanisms might account for the connection between obesity and sexual dysfunction. Obesity is a condition of inflammation and chronic oxidative stress [40]. It was suggested that obesity is linked to endothelial dysfunction and increased serum concentrations of vascular inflammatory makers [41, 42]. The visceral adipose tissue can secrete biochemical modulators and proinflammatory factors, such as IL-6, TNF-α, angiotensinogen, angiotensin-converting enzyme and so on, which can be obviously associated with systemic and peripheral vascular inflammation and can subsequently lead to a decrease in NO synthase and NO activity, an increase in adhesion molecules, MCP-1 and M-CSF, finally causing endothelial dysfunction [43–45]. Moreover, the reduced adiponectin level has been confirmed to be connected with endothelial dysfunction [46, 47]. The endothelial dysfunction causes erectile dysfunction by influencing the structural integrity of the vascular bed in the penis and the progress of penile engorgement by reducing the blood flow of penile [48]. It can ameliorate endothelial function by increasing vasodilation in which the endothelium plays an important role, increasing the level of activation marks of endothelium and decreasing the level of proinflammatory factor when obese patients proceed to loss weights [49]. Furthermore, the sex hormones have considerable correlation with sexual dysfunction. It is reported that the abnormalities in sex hormone regulation and production are related to sexual dysfunction in men [50, 51]. It has been confirmed that androgens are essential in maintaining the libido and regulating erectile capacity in men [52]. But BMI is negatively associated with serum testosterone in some studies. It was reported that a reduction in free testosterone of 1.35 pg/mL and a reduction in total testosterone of 11.79 ng/dL could be expected while the weight increases 4.5 kg [53]. Previous studies found a positive relationship between estradiol levels and BMI index in men [54]. And the reduction of estradiol levels is thought to be associated with weight loss. Thus, the feedback inhibition on LH secretion is removed and the testosterone secretion is enhanced. This mechanism was supported by European Male Aging Study, in which a negative correlation between body weight and total testosterone, free testosterone and sex hormone-binding globulin (SHBG) was reported [55].

There are some limitations of our study. (1) Language bias might limit the generalizability of the findings. (2) Patient self-report is the current standard of male sexual function assessment in this study. (3) Included sample sizes are small. (4) The indexes for evaluating male’s sexual function, including IIEF score and BSFI score are simple, and are not both reported in all studies. (5) The length of follow-up is relatively short and long-term results are not available. Those limitations might lead to the analysis bias and influence the reliability of our study.

## Conclusions

We found that the bariatric surgery could be effective in improving male’s sexual function in obese individuals, with significantly improved IIEF scores and BSFI scores. These results are in accordance with the results of recent studies. With the limited number of studies evaluating the specific factor, including psychological parameter, PDE5i use, age and so on, and its influence on sexual function in obese individuals undergoing bariatric surgery, we could not make a comprehensive view on those factors. More well designed studies conducted on this topic is needed to confirm our findings.

## Data Availability

All data generated or analyzed during this study are included in this published.
